# Can Panoramic Radiography Reliably Detect Root Resorption From Impacted Maxillary Canines?

**DOI:** 10.7759/cureus.107602

**Published:** 2026-04-23

**Authors:** Mustafa A Badi, Peter Gakunga, Hassem Geha, Funda Yilmaz, Marcel Noujeim

**Affiliations:** 1 Oral and Maxillofacial Radiology, Temple University, Maurice H. Kornberg School of Dentistry, Philadelphia, USA; 2 Orthodontics, Independent Consulting, San Antonio, USA; 3 Oral and Maxillofacial Radiology, University of Texas Health Science Center at San Antonio, San Antonio, USA; 4 Endodontics, Division of Oral Maxillofacial Radiology, Touro College of Dental Medicine, Hawthorne, USA; 5 Oral and Maxillofacial Radiology, Advanced Imaging Diagnostics, San Antonio, USA

**Keywords:** cone-beam computed tomography, diagnostic accuracy, impacted tooth, maxillary canine, observer performance, panoramic radiography, root resorption

## Abstract

Background and objective

Panoramic radiography (PR) remains widely used for the initial assessment of impacted maxillary canines; however, its diagnostic accuracy for detecting associated root resorption has not been well quantified across different observer categories. This study aimed to evaluate the diagnostic accuracy of PR for detecting root resorption associated with impacted maxillary canines, using cone beam CT (CBCT) as the reference standard.

Materials and methods

A total of 51 patients (71 impacted canine sites) with paired panoramic radiographs and CBCT images were retrospectively evaluated. Six observers - two oral and maxillofacial radiology residents, two orthodontic residents, and two general dentists - independently assessed panoramic radiographs for the presence, severity, and location of root resorption using a 5-point confidence scale. Two board-certified oral and maxillofacial radiologists established the reference standard findings through consensus evaluation of CBCT images. Diagnostic accuracy was assessed using receiver operating characteristic (ROC) curve analysis.

Results

Root resorption was present in 39.4% of sites (28/71) based on CBCT evaluation. The overall area under the ROC curve (AUC) for PR was 0.659 (95% confidence interval (CI): 0.615-0.703). At the ≥4 threshold, overall sensitivity was 63.1%, and specificity was 59.3%. Severity classification demonstrated fair agreement for apical resorption (κ = 0.22) and slight agreement for lateral resorption (κ = 0.05). Central incisors were affected in 37.1% of resorption cases.

Conclusions

Based on our findings, PR demonstrates limited yet clinically useful diagnostic accuracy for detecting root resorption associated with impacted maxillary canines. It retains its value as an initial screening tool.

## Introduction

Maxillary canines are the second most commonly impacted teeth after third molars, with prevalence rates of 1 to 3% in the general population and up to 23.5% in orthodontic practices. Root resorption of adjacent teeth, particularly lateral incisors, represents the most significant complication associated with impacted maxillary canines [1-4]. Depending on severity, root resorption may complicate treatment planning, compromise tooth prognosis, or necessitate extraction. Early detection and accurate grading of the extent of resorption are therefore critical for optimal treatment decision making [5]. External apical root resorption can also occur as a consequence of orthodontic treatment, further emphasizing the importance of an accurate baseline assessment before intervention [6].

Radiographic assessment of impacted canines has traditionally relied on two-dimensional (2D) imaging modalities, including panoramic, periapical, and occlusal radiographs. Panoramic radiography (PR) remains the most widely utilized initial imaging modality due to its broad anatomical coverage, relatively low radiation dose, and widespread availability [7]. However, the inherent limitations of 2D projection geometry, including superimposition of anatomical structures, geometric distortion, and lack of bucco-palatal information, compromise its diagnostic accuracy in detecting subtle pathology such as early root resorption [8].

Cone beam CT (CBCT) has demonstrated superior diagnostic accuracy for detecting root resorption compared to conventional radiography [9-14]. Hajeer et al. [12] demonstrated in an in-vitro study that CBCT-based interpretations significantly outperformed conventional radiography for localizing impacted canines. Alfailany et al. [13] further validated these findings in vivo, comparing imaging assessments against the gold standard of direct surgical visualization, and reported that CBCT provided significantly more accurate localization and root resorption detection than 2D methods. A recent systematic review and meta-analysis by Peralta-Mamani et al. [14] reported that CBCT demonstrated significantly higher detection rates than PR (odds ratio (OR): 0.14, 95% confidence interval (CI): 0.06-0.33), confirming the substantial diagnostic advantage of three-dimensional imaging. International guidelines, including those from the American Academy of Oral and Maxillofacial Radiology (AAOMR) and the European SEDENTEXCT consortium, recommend CBCT when 2D imaging provides insufficient diagnostic information for treatment planning [15,16].

Receiver operating characteristic (ROC) curve analysis is a well-established method for evaluating diagnostic test performance. The area under the ROC curve (AUC) provides a threshold-independent measure of discriminative ability, where an AUC of 1.0 represents perfect discrimination and 0.5 represents chance performance [17].

Despite the established superiority of CBCT, access to this technology remains limited in many clinical settings worldwide. Equipment costs, infrastructure requirements, and a shortage of trained professionals restrict CBCT availability, particularly in developing countries [18]. Furthermore, radiation dose considerations, especially pertinent in pediatric and adolescent populations who constitute the majority of patients with impacted canines, support judicious use of 3D imaging [19]. Understanding the diagnostic capabilities and limitations of PR, therefore, remains clinically relevant for appropriate case selection and CBCT referral decisions. In light of this, the purpose of this diagnostic accuracy study was to evaluate PR performance for detecting root resorption associated with impacted maxillary canines, using CBCT as the reference standard. Secondary objectives included comparison of diagnostic performance across different observer categories and assessment of PR accuracy for determining resorption severity and canine localization.

## Materials and methods

Study population and design

This retrospective cross-sectional diagnostic accuracy study followed the Standards for Reporting Diagnostic Accuracy Studies (STARD) guidelines. The study was approved by the Institutional Review Board of the University of Texas Health Science Center at San Antonio (Protocol #HSC20070348H). Informed consent was waived due to the retrospective nature of the study and use of de-identified imaging data.

Records of patients aged 10 years and older referred to the graduate oral and maxillofacial radiology clinic for CBCT evaluation of impacted or ectopically erupting maxillary canines between January 2006 and December 2011 were reviewed. The availability of paired PR and CBCT imaging reflects standard clinical practice at our institution, where patients referred for CBCT evaluation of impacted canines typically had recent panoramic radiographs from referring practitioners.

The inclusion criteria were as follows: (1) unilateral or bilateral impacted or ectopically erupting maxillary canines with adjacent lateral incisors present; (2) availability of both panoramic radiograph and CBCT volume acquired within six months of each other. The exclusion criteria were as follows: extensive restorations obscuring the region of interest, current or previous orthodontic treatment, missing teeth adjacent to impacted canines, and pathology in the region of interest.

**Figure 1:**
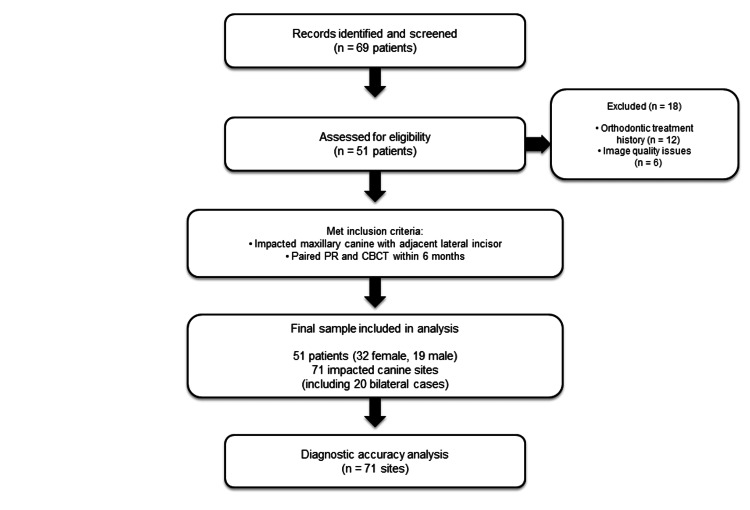
Flow diagram depicting patient selection Flow diagram of patient selection following the Standards for Reporting Diagnostic Accuracy Studies (STARD) guidelines. Of the 69 patients initially identified, 18 were excluded, yielding a final sample of 51 patients with 71 impacted canine sites PR: panoramic radiography; CBCT: cone beam computed tomography

Of the 69 patients initially identified, 18 were excluded due to orthodontic treatment history or image quality degradation from extensive restorations. The process of sample selection and application of exclusion criteria to experimental groups is summarized in Figure 1. The final sample comprised 51 patients (32 females, 19 males) with 71 impacted maxillary canines, including 20 bilateral cases. The unit of analysis was defined as each impacted maxillary canine and its adjacent teeth evaluated as a single diagnostic site. The median interval between PR and CBCT acquisition was 21 days (interquartile range (IQR): 7-45 days); 89% of paired images were acquired within three months. This study was exploratory in nature, and a formal a priori power calculation was not performed. Post-hoc analysis indicated that with 71 diagnostic sites, 39.4% disease prevalence, and alpha = 0.05, the study had approximately 80% power to detect an AUC significantly different from 0.5.

Image acquisition

**Figure 2:**
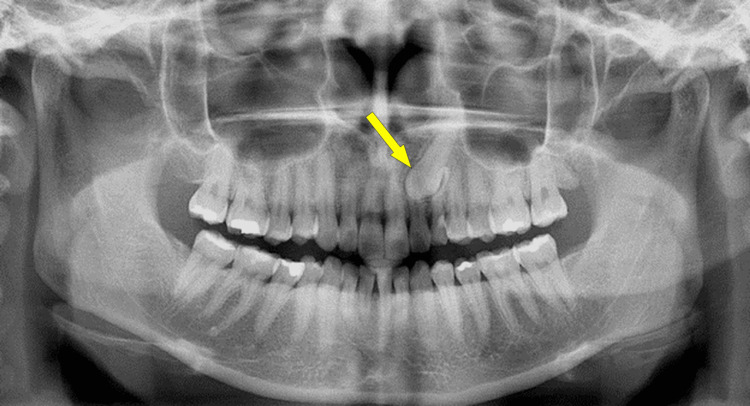
Panoramic radiograph Representative panoramic radiograph demonstrating an impacted maxillary canine (arrow)

Panoramic radiographs were acquired using a Planmeca Promax unit (Planmeca, Helsinki, Finland) with standard exposure parameters (66-70 kV, 8-10 mA, 18-second exposure time). Images were exported from MiPACS Dental Enterprise software (Medicor Imaging, Charlotte, NC) and displayed on a calibrated flat-panel monitor (1920 × 1080 resolution, 300 cd/m² luminance) under standardized viewing conditions with ambient lighting reduced to < 50 lux (Figure 2).

**Figure 3:**
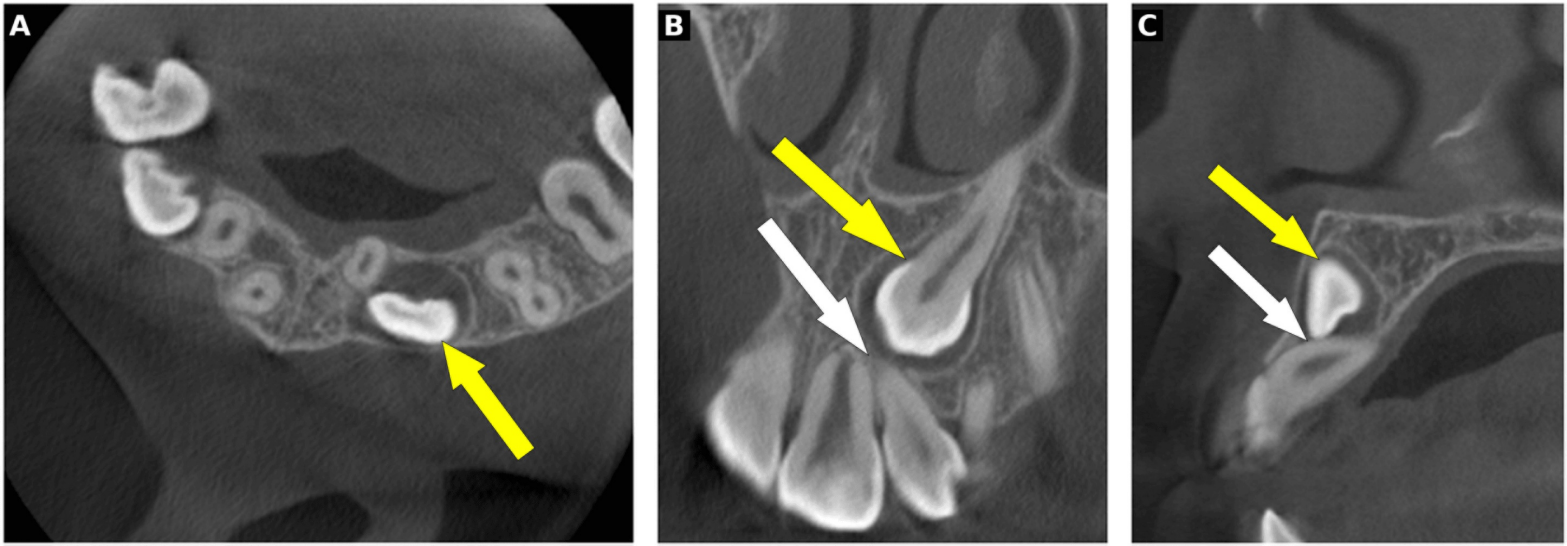
CBCT images: axial (A), coronal (B), and sagittal (C) views Representative CBCT images showing axial (A), coronal (B), and sagittal (C) views of an impacted maxillary canine (yellow arrow) with associated root resorption of the adjacent lateral incisor (white arrow) CBCT: cone beam computed tomography

CBCT volumes were acquired using an Accuitomo 3D unit (J. Morita, Kyoto, Japan) with 0.125 mm isotropic voxel size. The field of view was selected based on clinical indication: 40×40 mm for unilateral cases and 60 × 60 mm for bilateral cases. Volumes were viewed using i-Dixel One Data viewer software (J. Morita, Kyoto, Japan) with multiplanar reconstruction capability (Figure 3).

Image evaluation

Two board-certified oral and maxillofacial radiologists (with 8 and 12 years of post-residency experience, respectively) independently evaluated all CBCT volumes to establish the reference standard. These radiologists did not participate in the PR evaluation phase. They assessed (1) the presence of root resorption on teeth adjacent to impacted canines; (2) severity and location of resorptive lesions; and (3) bucco-palatal position of impacted canines. Lateral surface resorption was graded using the CT-based classification described by Ericson and Kurol [2], specifically developed for canine-induced resorption. Apical resorption severity was graded using the Malmgren index [20], a widely accepted system for quantifying external apical root resorption. Disagreements between the two radiologists were resolved by consensus discussion.

Six observers evaluated the panoramic radiographs: two oral and maxillofacial radiology residents (PGY-2 and PGY-3), two orthodontic residents (PGY-2 and PGY-3), and two general dentists (with three and five years of clinical experience). None of the PR observers had prior formal training in CBCT interpretation. All observers were blinded to patient clinical records and CBCT findings throughout the study.

Before evaluation, all observers completed a two-hour calibration session that included (1) didactic review of root resorption classification systems; (2) review of 10 example cases demonstrating the spectrum of resorption severity; (3) practice scoring with immediate feedback; and (4) group discussion to establish consensus on classification criteria. Following calibration, observers evaluated randomized panoramic radiographs in two sessions separated by one week. Images were presented in a different random order for each observer using computer-generated randomization. Twenty radiographs (28% of the sample) were randomly selected and re-evaluated in the second session for intra-observer reliability assessment; observers were not informed which images were repeated.

Observers recorded (a) the presence or absence of root resorption using a 5-point confidence scale (1 = definitely absent, 2 = probably absent, 3 = unsure, 4 = probably present, 5 = definitely present); (b) location of resorption (apical, middle, or cervical third); (c) severity of resorption (apical and lateral aspects separately); and (d) bucco-palatal position of impacted canines (buccal, palatal, or mid-alveolus).

Statistical analysis

Diagnostic accuracy was assessed using ROC analysis. AUC was calculated for each observer and for observer categories using the trapezoidal method. Confidence intervals for AUC were computed using DeLong’s method. Sensitivity and specificity were calculated at two clinically relevant thresholds: grades ≥4 as positive (high confidence) and grades ≥3 as positive (including uncertain cases). Observer agreement was assessed using Cohen’s kappa for intra-observer reliability and Fleiss’ kappa for inter-observer agreement. Weighted kappa (quadratic weights) was calculated for ordinal severity scales. Kappa values were interpreted according to Landis and Koch [21]: < 0.00: poor, 0.00-0.20: slight, 0.21-0.40: fair, 0.41-0.60: moderate, 0.61-0.80: substantial, 0.81-1.00: almost perfect.

Comparison of diagnostic performance across observer categories was performed using analysis of variance (ANOVA) with Tukey-Kramer post-hoc testing for multiple comparisons. Given the inclusion of bilateral cases, we performed a sensitivity analysis using generalized estimating equations (GEE) with an exchangeable correlation structure to account for within-patient clustering; results were consistent with the primary analysis. A p-value < 0.05 was considered statistically significant. Analyses were performed using SAS software version 9.3 (SAS Institute Inc., Cary, NC).

## Results

Reference standard findings

Patient ages ranged from 10 to 71 years (mean: 24.7, median: 16, standard deviation (SD): 16.6). Females comprised 62.7% (32/51) of subjects, and 58.8% (30/51) were aged 10-20 years. Of the 71 impacted canines, 36 (50.7%) were palatally positioned, 20 (28.2%) were in mid-alveolus, and 15 (21.1%) were buccally positioned based on CBCT reference standard evaluation.

Root resorption was detected on CBCT at 28 of 71 sites (39.4%), affecting 35 teeth. Lateral incisors were most commonly involved (n = 19, 54.3%), followed by central incisors (n = 13, 37.1%) and premolars (n = 3, 8.6%). Multiple teeth were affected in eight cases (28.6%), with an average of 1.25 teeth involved per resorption site (Table 1). Sixteen of 28 resorption sites (57.1%) occurred in patients aged 10-20 years. Regarding severity on CBCT, 16 sites exhibited severe resorption, three moderate, and nine mild. Resorption was most commonly located in the apical third. Buccally positioned canines demonstrated the highest resorption rate (60%, 9/15), compared to palatally positioned (38.9%, 14/36) and mid-alveolus (25%, 5/20) canines.

**Table 1:** Distribution of teeth affected by root resorption on CBCT reference standard Average teeth affected per resorption site: 1.25 CBCT: cone beam computed tomography

Distribution of teeth affected		
Tooth type	N	Percentage
Lateral incisors	19	54.30%
Central incisors	13	37.10%
Premolars	3	8.60%
Total teeth affected	35	100%
Pattern of involvement	N	Percentage
Single tooth	20	71.40%
Multiple teeth	8	28.60%
Total resorption sites	28	100%

Diagnostic accuracy

Intra-observer agreement for PR evaluation was moderate (mean κ = 0.51, range: 0.42-0.61), with no significant difference among observer categories (p = 0.83) (Table 2). Inter-observer agreement using the five-category confidence scale was slight (κ = 0.16). Using dichotomized scales, inter-observer agreement improved to fair (κ = 0.28 for grades ≥3 positive; κ = 0.32 for grades ≥4 positive).

**Table 2:** Intra-observer agreement (kappa) by observer category Analysis of variance: p = 0.83 for the difference among observer categories. Interpretation: moderate agreement (Landis & Koch criteria) CI: confidence interval

Observer category	Observer	Kappa (κ)	95% CI
Oral radiologists	1	0.48	0.31-0.65
	2	0.52	0.35-0.69
	Mean	0.5	—
Orthodontists	1	0.42	0.25-0.59
	2	0.55	0.38-0.72
	Mean	0.49	—
General dentists	1	0.61	0.44-0.78
	2	0.48	0.31-0.65
	Mean	0.55	—
Overall	—	0.51	0.42-0.61

**Figure 4:**
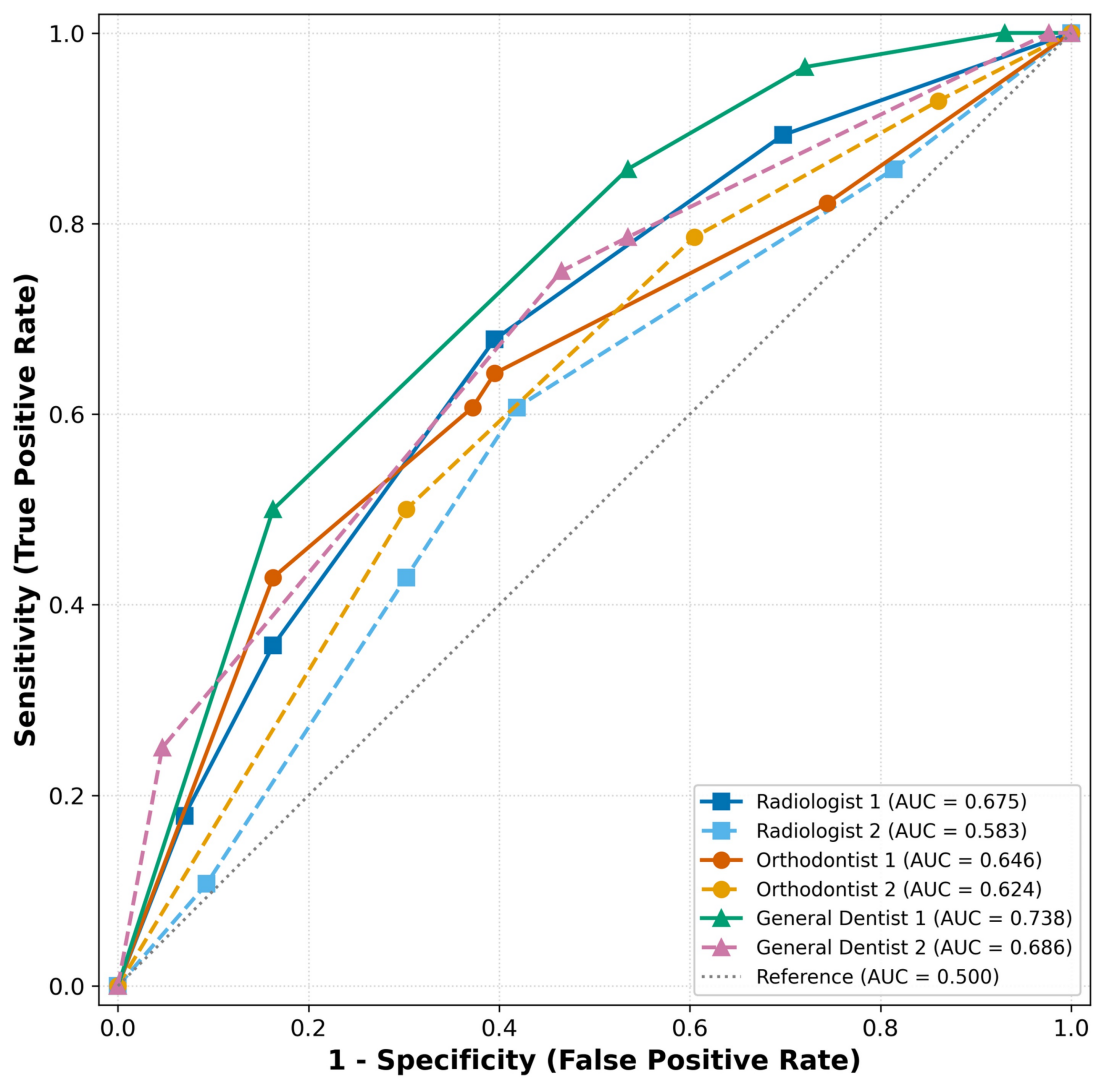
ROC analysis curve ROC curves for the detection of root resorption associated with impacted maxillary canines by six observers using panoramic radiography. Area under the curve values are shown for each observer. The diagonal dotted line represents chance performance (area under the curve = 0.500) ROC: receiver operating characteristic

The overall AUC for detecting root resorption on PR was 0.659 (95% CI: 0.615-0.703) (Figure 4). There was no statistically significant difference in AUC among the six individual observers (p = 0.17) or among observer categories (Table 3). General dentists achieved the highest mean AUC (0.712, 95% CI: 0.650-0.774), followed by orthodontists (0.635, 95% CI: 0.573-0.697) and radiologists (0.629, 95% CI: 0.567-0.691), even though these differences were not statistically significant (p = 0.26).

**Table 3:** AUC by observer and observer category Analysis of variance: p = 0.17 for difference among individual observers; p = 0.26 for difference among observer categories AUC: area under the ROC curve; ROC: receiver operating characteristic; CI: confidence interval

Observer category	Observer	AUC	95% CI
Oral radiologists	1	0.612	0.521-0.703
	2	0.646	0.556-0.736
	Mean	0.629	0.567-0.691
Orthodontists	1	0.658	0.568-0.748
	2	0.612	0.521-0.703
	Mean	0.635	0.573-0.697
General dentists	1	0.734	0.649-0.819
	2	0.69	0.602-0.778
	Mean	0.712	0.650-0.774
Overall	—	0.659	0.615-0.703

Using grades ≥4 as the positive threshold, the overall sensitivity was 63.1% (95% CI: 55.8-70.4) and specificity was 59.3% (95% CI: 50.0-68.6). Sensitivity differed significantly among observer categories (p = 0.04): general dentists (80.4%) demonstrated higher sensitivity than radiologists (39.3%), with orthodontists intermediate (69.6%). Specificity did not differ significantly among groups (p = 0.16) (Table 4). Using grades ≥3 as positive, sensitivity increased to 74.4% while specificity decreased to 48.8%, with no significant differences among observer categories. Analysis of response distribution demonstrated systematic differences in confidence level utilization. Oral and maxillofacial radiologists used grade 3 (“unsure”) responses in 20.4% of assessments, compared with 10.6% for general dentists and 1.4% for orthodontists.

**Table 4:** Sensitivity and specificity by observer category at two thresholds Threshold ≥ 4: analysis of variance: p = 0.04 for sensitivity difference; p = 0.16 for specificity difference among categories. Threshold ≥ 3: analysis of variance: p = 0.62 for sensitivity difference; p = 0.38 for specificity difference among categories CI: confidence interval

Sensitivity and specificity				
Threshold ≥ 4 (high confidence positive)				
Observer category	Sensitivity (%)	95% CI	Specificity (%)	95% CI
Oral radiologists	39.3	28.1-51.5	74.4	63.9-82.9
Orthodontists	69.6	57.5-79.7	55.8	44.1-66.9
General dentists	80.4	69.2-88.4	47.7	36.3-59.3
Overall	63.1	55.8-70.4	59.3	50.0-68.6
Threshold ≥ 3 (including uncertain cases)				
Observer category	Sensitivity (%)	95% CI	Specificity (%)	95% CI
Oral radiologists	71.4	59.6-81.0	48.8	37.4-60.4
Orthodontists	73.2	61.6-82.5	53.5	41.8-64.8
General dentists	78.6	67.4-86.9	44.2	33.0-55.9
Overall	74.4	68.1-80.0	48.8	40.5-57.2

Severity classification and localization

For severity grading on PR, correct classification occurred in 78.1% of sites without resorption but only 21-25% of sites with mild, moderate, or severe resorption (Tables 5, 6). Combined apical and lateral severity assessment showed correct classification in 58.5% of non-resorption cases and 11-26% of cases with resorption present. Overall agreement with reference standard severity was fair for apical resorption (κ = 0.22, weighted κ = 0.28) and slight for lateral resorption (κ = 0.05, weighted κ = 0.09).

**Table 5:** Severity classification accuracy: PR assessment vs. CBCT reference standard (apical resorption) Kappa (κ) = 0.22 (fair agreement); weighted kappa = 0.28 PR: panoramic radiography; CBCT: cone beam computed tomography

CBCT reference	PR assessment: correct (%)	PR assessment: incorrect (%)
No resorption (n = 43)	78.1	21.9
Mild (n = 9)	22.2	77.8
Moderate (n = 3)	25	75
Severe (n = 16)	21.4	78.6

**Table 6:** Severity classification accuracy: PR assessment vs. CBCT reference standard (lateral resorption) Kappa (κ) = 0.05 (slight agreement); weighted kappa = 0.09 PR: panoramic radiography; CBCT: cone beam computed tomography

CBCT reference	PR assessment: correct (%)	PR assessment: incorrect (%)
No resorption (n = 43)	58.5	41.5
Mild (n = 12)	16.7	83.3
Moderate (n = 8)	12.5	87.5
Severe (n = 8)	25	75

**Figure 5:**
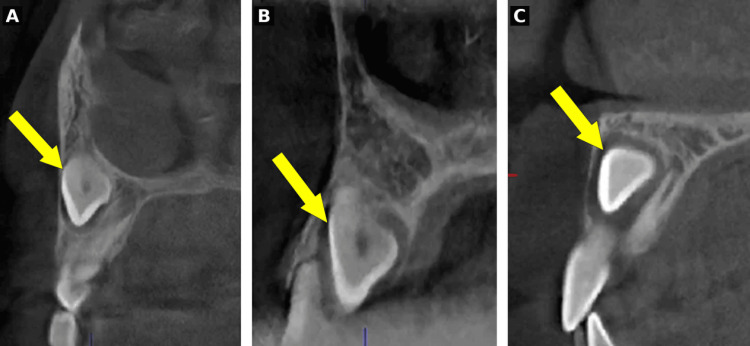
CBCT sagittal views CBCT sagittal views illustrating canine position classification (arrows): (A) buccal, (B) palatal, and (C) mid-alveolus impaction CBCT: cone beam computed tomography

Bucco-palatal canine position was correctly identified on PR in only 43% of cases overall (Figure 5). Detection was significantly better for palatally positioned canines (68.5%) compared to buccally positioned canines (22.2%; p = 0.02) (Table 7). Diagnostic performance varied by tooth type (Table 8). Sensitivity for detecting resorption was highest for central incisors (75.0%, 95% CI: 64.8-83.0), followed by lateral incisors (61.4%, 95% CI: 52.2-69.8), and lowest for premolars (33.3%, 95% CI: 16.3-56.3). The higher sensitivity for central incisors compared to lateral incisors may reflect the more severe resorption typically present when central incisors are involved, making detection on PR more likely.

**Table 7:** Bucco-palatal localization accuracy by canine position ^*^P-value for comparison between palatal vs. buccal position (Tukey-Kramer test) CBCT: cone beam computed tomography; PR: panoramic radiography

CBCT position	N	Correct on PR (%)	P-value
Palatal	36	68.5	—
Mid-alveolus	20	35	—
Buccal	15	22.2	—
Overall	71	43	0.02^*^

**Table 8:** Per-tooth diagnostic performance (sensitivity) by tooth type Note: higher sensitivity for central incisors may reflect more severe resorption typically present when central incisors are involved, making detection on PR more likely CI: confidence interval; PR: panoramic radiography

Tooth type	N (with resorption)	Sensitivity (%)	95% CI
Central incisors	14	75	64.8-83.0
Lateral incisors	19	61.4	52.2-69.8
Premolars	3	33.3	16.3-56.3

## Discussion

This diagnostic accuracy study evaluated PR performance for detecting root resorption associated with impacted maxillary canines, using CBCT as the reference standard. The overall AUC of 0.659 indicates limited but clinically useful diagnostic accuracy: performance meaningfully better than chance (0.5) but well below the excellent discrimination (>0.9) that would support definitive diagnosis. These findings are consistent with previous studies and the recent meta-analysis by Peralta-Mamani et al. [9,14].

It is essential to clarify the study design: this investigation evaluated PR as an index test against CBCT as the reference standard, rather than as a direct comparison of two equivalent diagnostic modalities. CBCT was selected as the reference standard because it represents the best available non-invasive method for detecting root resorption, although we acknowledge that histological examination would constitute the true gold standard, as validated by Kadesjö et al. [22]. Recent studies continue to confirm the diagnostic value of CBCT for detecting apical root resorption in various clinical contexts [23]. These findings quantify the performance clinicians can expect from PR when CBCT findings are considered the clinical truth.

Our findings align with Hajeer et al.’s [12] in-vitro study demonstrating superior CBCT performance for impacted canine assessment. While their controlled laboratory conditions differ from our clinical sample, both studies confirm the fundamental limitations of 2D imaging for this application. Alfailany et al. [13] provided important in-vivo validation using surgical exposure as the gold standard, reporting that 2D methods significantly underestimated root resorption compared to direct visualization. Our AUC of 0.659 is consistent with their reported diagnostic accuracy metrics and supports the conclusion that PR, while useful for screening, cannot replace 3D imaging for definitive diagnosis.

The observed resorption prevalence (39.4%) aligns with previous CBCT-based studies reporting rates of 30-50% [24,25]. Notably, central incisors comprised over one-third of affected teeth (37.1%), highlighting that resorption risk extends beyond the immediately adjacent lateral incisor. This finding has important clinical implications: comprehensive radiographic evaluation should include all teeth in proximity to impacted canines, not solely lateral incisors. The observation that multiple teeth were involved in nearly one-third of cases (28.6%) underscores the potential for widespread resorptive damage when impacted canines remain untreated.

An interesting finding was the significant difference in sensitivity among observer categories, with general dentists demonstrating higher sensitivity (80.4%) than oral radiologists (39.3%). Analysis of response patterns revealed that radiologists used “unsure” responses significantly more frequently (20.4%) compared with general dentists (10.6%) and orthodontists (1.4%). This conservative approach likely reflects greater familiarity with 2D imaging limitations and awareness of CBCT’s superior diagnostic capability. This phenomenon, where specialist knowledge may paradoxically reduce diagnostic confidence on conventional imaging, has been reported in other contexts [26]. Radiologists, knowing what CBCT can reveal, may be more hesitant to render definitive judgments on PR. From a clinical standpoint, the higher sensitivity of general dentists (albeit with lower specificity) may be advantageous for screening purposes, where the primary goal is avoiding missed pathology.

The poor performance in severity classification (fair to slight kappa agreement) and bucco-palatal localization (43% accuracy) reflects fundamental limitations of 2D projection geometry, consistent with findings by Lai et al. [27]. Ghost imaging, geometric magnification, and anatomical superimposition inherent to the panoramic technique preclude accurate assessment of resorption depth and three-dimensional relationships.

While CBCT is recognized as the clinical reference standard for evaluating impacted canines and associated root resorption, access remains restricted in many settings globally [18]. Our findings support PR’s continued role as an initial screening tool while providing specific performance metrics to guide clinical decision-making. Based on our results, CBCT is indicated when PR findings are suspicious or uncertain, and when the results would change clinical management, such as determining canine localization, assessing resorption extent, or informing surgical planning. This approach aligns with the AAOMR and European guidelines emphasizing justification and ALARA ("as low as reasonably achievable") principles [15,16]. Recent prospective studies confirm that supplemental CBCT frequently changes treatment plans based on 2D radiography alone [28], and that specific 2D radiographic features can help predict when CBCT is justified [29]. The position of impacted canines relative to adjacent incisors significantly influences resorption incidence [30].

This study has several limitations. Firstly, the sample was derived from a referral-based population with clinical suspicion of canine impaction, potentially representing more complex cases than encountered in general practice. Second, while we allowed up to six months between PR and CBCT acquisition, 89% of paired images were obtained within three months (median: 21 days). Third, four of six PR observers were residents, which may limit generalizability to experienced practitioners. Fourth, no formal a priori power calculation was performed; this study was exploratory in nature, and the post-hoc power analysis (approximately 80% power to detect an AUC significantly different from 0.5) should be interpreted with caution, as post-hoc calculations do not substitute for prospective sample size planning. Fifth, the inclusion of bilateral cases introduces potential within-patient correlation, though GEE analysis accounting for clustering produced consistent results. Sixth, while CBCT served as our reference standard, histological examination would represent the true gold standard [22].

## Conclusions

PR demonstrates limited diagnostic accuracy for detecting root resorption associated with impacted maxillary canines, with an overall AUC of 0.659. Severity classification and bucco-palatal canine localization showed poor agreement with CBCT reference findings. Importantly, root resorption affected central incisors in over one-third of cases and involved multiple teeth in nearly one-third of resorption sites, emphasizing the need for a comprehensive evaluation of all adjacent teeth. While CBCT serves as the reference standard for clinical diagnosis, PR may serve as an initial screening tool, particularly in settings with limited CBCT access or when radiation dose considerations favor two-dimensional imaging. However, its moderate diagnostic performance and poor agreement in severity and localization assessment underscore the need for CBCT when an accurate diagnosis is required for clinical decision-making. CBCT is indicated when panoramic findings are suspicious or uncertain and when the results are expected to influence clinical management, consistent with ALARA principles and international guideline recommendations.
